# Central Plasticity of Cutaneous Afferents Is Associated with Nociceptive Hyperreflexia after Spinal Cord Injury in Rats

**DOI:** 10.1155/2019/6147878

**Published:** 2019-11-19

**Authors:** Hyun Joon Lee, Patrick S. Malone, Jumi Chung, Jason M. White, Natalee Wilson, Jason Tidwell, Keith E. Tansey

**Affiliations:** ^1^Departments of Neurology and Physiology, Emory University School of Medicine, Atlanta GA 30322, USA; ^2^Department of Biomedical Engineering, Georgia Institute of Technology/Emory University, Atlanta, GA 30322, USA; ^3^Spinal Cord Injury Clinic, Atlanta VA Medical Center, Atlanta GA 30033, USA

## Abstract

Electrical stimulations of dorsal cutaneous nerves (DCNs) at each lumbothoracic spinal level produce the bilateral cutaneus trunci muscle (CTM) reflex responses which consist of two temporal components: an early and late responses purportedly mediated by A*δ* and C fibers, respectively. We have previously reported central projections of DCN A and C fibers and demonstrated that different projection patterns of those afferent types contributed to the somatotopic organization of CTM reflex responses. Unilateral hemisection spinal cord injury (SCI) was made at T10 spinal segments to investigate the plasticity of early and late CTM responses 6 weeks after injury. Both early and late responses were drastically increased in response to both ipsi- and contralateral DCN stimulations both above (T6 and T8) and below (T12 and L1) the levels of injury demonstrating that nociceptive hyperreflexia developed at 6 weeks following hemisection SCI. We also found that DCN A and C fibers centrally sprouted, expanded their projection areas, and increased synaptic terminations in both T7 and T13, which correlated with the size of hemisection injury. These data demonstrate that central sprouting of cutaneous afferents away from the site of injury is closely associated with enhanced responses of intraspinal signal processing potentially contributing to nociceptive hyperreflexia following SCI.

## 1. Introduction

Spinal cord injury (SCI) often results in devastating pain that largely impacts the quality of life in patients. A longitudinal study for 5 years demonstrated that somatic pain is the most common type of pain in SCI patients regardless of the type of injury, the injury severity, and the onset time of pain-related symptoms [1]. This implies that damage at the spinal cord of any size may cause early or late changes in nociceptive signal pathways which result in persistently enhanced pain sensitivity, e.g., nociceptive hyperreflexia. This maladaptive plasticity appears to be a consequence of central changes that include loss of supraspinal inhibitory control [2], death of inhibitory propriospinal interneurons [3], reduced GABA (GAD65) synthesis [4], interrupted chloride equilibrium in spinal neurons [5], and sprouting of nociceptive afferents [6–8]. These changes mostly occur at the level of SCI but may expand away, both above and below the level of injury, generating hyperreflexia in nociceptive circuitries that are not directly affected by that injury.

Nociceptive afferent sprouting distal to the site of SCI is related to activation of their intrinsic growth capability. Small and medium-sized, but not large diameter, dorsal root ganglia (DRG) neurons dissociated from uninjured segments after T10 contusion SCI are capable of elongating their neurites *in vitro* [9]. Nociceptive afferent sprouting away from the site of SCI has been extensively evidenced with increased immunoreactivity for calcitonin gene-related peptide (CGRP) expressed in peptidergic afferents [2, 6–8, 10] and for isolectin B4 (IB4) that binds to nonpeptidergic afferents [6, 11]. Sprouting of those C fiber populations is related to different types of pathophysiology following SCI: nociceptive hyperreflexia with IB4 binding afferents [11], CGRP bearing afferents [7], and their increased overlap [6] in superficial laminae and autonomic dysreflexia with CGRP bearing afferents in deeper laminae [8, 10, 12].

**Figure 1:**
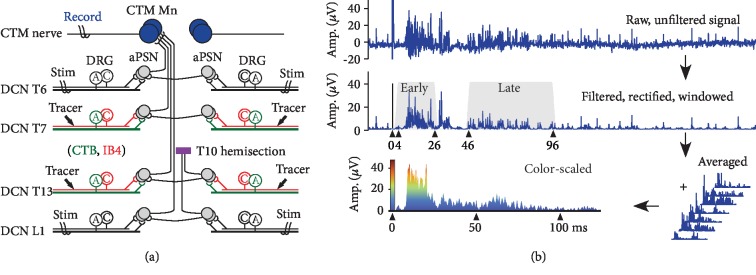
Wiring diagram of the cutaneus trunci muscle (CTM) reflex and CTM neurogram signal processing. (a) A lateral hemisection injury was made at the T10 spinal level on the right side of animals (purple box). CTM motor responses were evoked by electrical stimulations (Stim) given at each dorsal cutaneous nerve (DCN) level, above (T6 and T8) and below (T12 and L1) the level of injury (T10), of each side of rats 6 weeks after injury as well as in uninjured normal controls. In other groups of those normal and injured animals, axon tracers were injected to T7 and T13 DCNs: cholera toxin subunit B (CTB, green) for myelinated A fibers and isolectin B4 (IB4, red) for unmyelinated C fibers. DRG = dorsal root ganglion; aPSN = ascending propriospinal neuron; MN = motoneuron. (b) CTM neurograms were evoked at 5 mA that evokes both early and late responses mediated by A*δ* and C fibers, respectively. Raw recording data were filtered, rectified, time-windowed for early (3.5–25.5 ms) and late (45.5-95.5 ms) responses from the stimulation onset. CTM responses were averaged across animals, smoothed, and color-scaled based on amplitudes (*μ*V).

Despite growing knowledge of roles for afferent plasticity in pain development, relatively less is known about how the different types of nociceptive afferents (A*δ* vs. C fibers), the extent of their sprouting, and the manner in which they are activated contribute to the generation of SCI-induced hyperreflexia. To address these questions, a quantifiable animal model of spinal signal processing that is assessable physiologically and anatomically is required. One such model is the cutaneus trunci muscle (CTM) reflex in rats. In addition to the lack of CTM, humans have no directly relevant reflex. The most comparable reflex is the abdominal or erector spinae skin reflexes [13]. The CTM reflex (Figure 1(a)) consists of three neuronal components: dorsal cutaneous nerves (DCNs) from each cervical to lumbosacral spinal segments, ascending propriospinal interneurons, and the CTM motoneurons in the cervicothoracic junction [14–19]. Electrical stimulation of DCNs evokes early and late CTM responses (Figure 1(b)) mediated by A*δ* and C fibers [15, 17, 19, 20]. The CTM reflex shows unique spatial features, e.g., the multisegmental organization of DCNs and the somatotopic arrangement of CTM motoneurons [18], although little is known about the propriospinal interneurons that connect the afferents and the motoneurons. Based on these previous findings, the CTM reflex is an attractive model for investigating residual connections and plasticity after SCI as well as for developing therapeutic interventions to SCI [15, 17].

In our previous studies in normal rats [20], it was demonstrated that evoked CTM neurogram responses, both early and late responses, were organized in a somatotopic manner depending on which spinal segmental DCNs were stimulated. We also labeled DCN afferent subtypes with cholera toxin subunit B (CTB) for myelinated A fibers and IB4 for unmyelinated C fibers at the T7 and T13 spinal levels. Central projection patterns of DCN afferents varied in systematic ways, e.g., projection area and spatial distribution of IB4^+^ C fibers, between T7 and T13 contributing to the somatotopic organization of physiological responses of the CTM reflex. Based on these connections between anatomy and physiology of this nociceptive reflex in the normal condition, we hypothesized that traumatic insults such as SCI trigger sprouting of nociceptive afferent limbs of this neural circuitry resulting in pathophysiological hyperreflexia measured as increased size of evoked CTM reflex responses. We further hypothesized that central changes in different DCN afferent subtypes, CTB^+^ A vs. IB4^+^ C fibers, selectively contribute to different temporal parts of CTM reflex responses, early vs. late responses, respectively.

## 2. Materials and Methods

### 2.1. Animals

Animals were kept in a regular 12-hour light-dark cycle, and *ad libitum* diet was provided. All animal procedures and postoperative care were approved by the Institutional Animal Care and Use Committee at Emory University School of Medicine.

### 2.2. Hemisection Spinal Cord Injury

Female Long-Evans rats (200-250 g, *n* = 15, Charles River Laboratories, Wilmington, MA) were anesthetized with an intraperitoneal injection of Ketamine (75 mg/kg, Bioniche, Morgantown, WV) mixed with Dexmedetomidine (0.25 mg/kg, Dexdomitor, Pfizer, New York, NY). The midthoracic back was shaved and sterilized with betadine and isopropyl ethanol swaps. Animals were kept on a heating pad regulated by a controller (TCAT-2DF, Physitemp Instruments, Clifton, NJ) with a rectal probe, and body temperature was maintained at 37°C during surgery. An incision was made along the median line of the back skin, and connective tissues were spread. Thoracic T9 vertebrate was identified by counting spinous processes from T2 and freed from surrounding muscles. Laminectomy was made at T9 vertebrate to expose the T10 spinal cord. A microscalpel was inserted straight down through the median line of the spinal cord, and a lateral hemisection was made to the right side of the animal. The incised dura mater remained open. Cut muscles were sutured, and the skin incision was stapled. Animals were recovered from anesthesia with a subcutaneous injection of atipamezole (2 mg/kg, Antisedan, Orion Pharma, Finland). Injured rats received subcutaneous injections of saline (0.5 ml) twice daily for 2 days and enrofloxacin (Baytril, 2.5 mg/kg, Bayer, Shawnee Mission, KS) once daily for 3 days. Soft cellulose bedding was used for a week. Injured rats were subjected to bladder expression twice daily until they recovered bladder function. Body weight was measured daily. Regular chow was provided inside the cage until they could reach the food container, and water was supplied with a long sipper tip. Surgical staples were removed 10 days after surgery.

### 2.3. Electrophysiology

A detailed surgical procedure of CTM neurogram recording in uninjured normal animals has been described in our previous report [20]. Six weeks after hemisection SCI, injured animals (*n* = 7) and their uninjured normal controls (*n* = 8) were prepared for a terminal electrophysiological experiment. Animals were anesthetized with an initial dose of pentobarbital (intraperitoneal injection, 50 mg/kg, Nembutal, Ovation Pharmaceuticals, Lincolnshire, IL). To maintain the anesthetic level, a supplemental injection (10% initial dose) was given every hour or when animals recovered the corneal reflex or responded to tail pinches. Animals were kept on a heating pad, and body temperature was maintained at 37°C using a thermal controller with a rectal probe during the rest of the experiment. The median back skin was shaved and incised between the base of the scapula and the ilium. DCNs were isolated from connective tissues on the body wall and cut distally at T6, T8, T12, and L1 spinal levels on both sides of the animal. A CTM motor nerve branch, typically the 3rd branch, was separated from muscles and cut distally only at the left side of the animal.

Animals were put on a stereotaxic frame, and the incised back skin was stretched with sutures to build a mineral oil bath. The cut CTM motor nerve branch was placed on a bipolar silver wire recording electrode, and neurograms were recorded continuously throughout the stimulation protocol. A DCN was placed on a bipolar stimulating electrode starting with the DCN at L1 on the left side of the animal. An electrical stimulation for 250 *μ*s at 5 mA that suffices to activate both A*δ* and C fibers was repeated in each stimulation train at different frequencies in the order of 1, 2, 5, and 10 Hz for 20 seconds which resulted in 20, 40, 100, and 200 total stimulations, respectively. An interval of 2 minutes without stimulation was used in between stimulation trains to allow CTM neurograms to return to their resting levels. After finishing 4 stimulation trains at the first DCN, within 2 minutes of the interval, the stimulating electrode was moved from the left to the right DCN at the same level and then rostrally in the order of L1, T12, T8, and T6. In several normal rats, various DCNs were stimulated at various stimulation frequencies again at the end of the recording session to confirm that these generated equivalent responses to the initial recordings. CTM neurograms were amplified 10,000 times using a differential amplifier (Model 3500, A-M Systems, Sequim, WA), and noises were removed with Humbug (Digitimer, Hertfordshire, UK). The data was recorded using a USB data acquisition board (NI-USB-6229, National Instruments, Austin, TX) and postprocessed using MATLAB (The MathWorks, Natick, MA). Briefly, the data was high pass filtered at 100 Hz, comb filtered at 60 Hz and 76.3 Hz, denoised (Wavelet denoising, Sym8 in MATLAB), and rectified. Neurogram responses at each stimulation number of the train were averaged across animals in each group, and their amplitudes were scaled in false colors (Figure 1(b)). The color-scaled amplitudes over 200 milliseconds from the stimulation onset were plotted as a horizontal line at each stimulation number (*y*-axis) of false color plots. To quantify evoked CTM responses, time windows were applied for early response, 3.5 to 25.5 milliseconds, and for late response, 45.5 to 95.5 milliseconds (Figure 1(b)) as determined in our previous studies in uninjured normal rats [20, 21]. Early and late responses were averaged across all stimulation at given frequencies and across all animals in each group. Changes in those responses were calculated relative to uninjured control animals, and percent changes 6 weeks after SCI were used.

### 2.4. Axon Tracer Injection

An additional group of rats with hemisection SCI (*n* = 8) underwent the same peripheral DCN injections of axon tracers as our previous studies in uninjured normal animals [20]. Three days before the 6-week time point following injury, rats were anesthetized with Ketamine (75 mg/kg)/Dexmedetomidine (0.25 mg/kg). The back skin was shaved, sterilized, and incised at the median line. T7 and T13 DCNs at both sides of animals were identified by counting from the first thoracic DCN branch. DCNs were separated from connective tissues along the lateral dorsal body wall proximally to the latissimus dorsi muscle. Isolectin B4 (IB4, Vector Laboratories, Burlingame, CA) was injected into T7 and T13 DCNs at one side of the animal, and cholera toxin subunit B (CTB, List Biological Laboratories, Campbell, CA) was injected on the other side (Figure 1(a)). Animals with right hemisection injury were divided into two subgroups to inject one label ipsilateral to the injury side (right) and the other to the contralateral side (left). This allowed to determine differences in DCN A and C fiber projections between injured and uninjured sides. A 30-guage needle was inserted 2-3 mm distal to the proximal entry for DCNs at the latissimus dorsi muscle. One microliter of 2% IB4 or CTB dissolved in PBS was injected with a micro syringe (75RN, Hamilton, Reno, NV) driven by a motorized pump (Model #310, Stoelting Co., Wood Dale, IL) at 1 *μ*l/min. The skin incision was stapled, and the animals were recovered from anesthesia with a subcutaneous injection of reversal agent (2 mg/kg, Antisedan).

### 2.5. Spinal Cord Tissue Processing

Six weeks after SCI, three days after tracer injections for the normal control group, rats underwent a transcardial perfusion with heparinized PBS (1 ml/L) and 4% paraformaldehyde (PFA). Spinal cord segments from T9 to T11 vertebral levels were resected from all injured animals for hemisection injury measurement. For animals injected with axon tracers, additional spinal cord segments were collected at vertebral levels from T6 to T8 and from T12 to L1. Resected spinal cord segments were postfixed in 4% PFA 24 hours, and cryoprotection was given with 10% and 30% sucrose solutions. Tissue segments were embedded in a molding with OCT medium (Optimal Cutting Temperature, Tissue-Tek, Torrance, CA) and frozen in 2-methylbutane on dry ice. Serial cryosectioning at a 20 *μ*m thickness produced longitudinal sections for hemisection injury measurement at T10 or cross sections for immunohistochemical analysis at T7 and T13.

### 2.6. Hemisection Injury Measurement

To measure percent hemisection injury for each animal, serial longitudinal spinal cord sections were stained with a 0.1% solution of Luxol fast blue in 95% alcohol and a 0.1% solution of Cresyl violet in deionized water. Sections were imaged using a light microscope with a 2x objective lens. Damaged spinal cord tissue was defined by the lack of myelination (Luxol fast blue staining) in the white matter, and the cytoarchitecture (Cresyl violet staining) of the scar tissue along which the damaged tissue was outlined. A reference line in a rostral-caudal axis was drawn at the edge of spinal cord sections on the uninjured side. A perpendicular line to the reference line was drawn to find the closest intersection with the damaged tissue outline and further extended to the tissue edge on the injured side of the cord. The length of this extended line from the intersection to the damaged tissue edge was measured as size of the injured tissue. A full length of the spinal cord tissue was measured from the reference line to the tissue edge on the other side at an adjacent noninjured tissue part. Percent hemisection (the size of injured tissue/the length of the spinal cord tissue ×100) was calculated on each serial longitudinal section at an interval of 100 *μ*m and averaged in each animal.

### 2.7. Immunohistochemistry and Microscopy

Cross sections at T7 and T13 were subjected to immunohistochemistry. Primary antibodies were used for IB4 (goat, Vector Laboratories, AS-2104, AB_2314660, 1 : 400), CTB (goat, List Biological Laboratories, 703, AB_10013220, 1 : 2500), VGLUT1 (rabbit, Synaptic Systems, 135302, AB_887877, 1 : 2000), CGRP (guinea pig, Peninsula Lab, T-5053, AB_1113068, 1 : 2000), and synaptophysin (mouse, Millipore, MAB5258, AB_95185, 1 : 2000). Cy3-conjugated anti-goat (705-165-147, AB_2307351, 1 : 400), Alexa Fluor 488-conjugated anti-goat (705-545-147, AB_2336933, 1 : 400), DyLight 488-conjugated anti-rabbit (711-485-152, AB_2492289, 1 : 400), Alexa Fluor 488-conjugated anti-guinea pig (706-545-148, AB_2340472, 1 : 100), Alexa Fluor 488-conjugated anti-mouse (715-545-150, AB_2340846, 1 : 400), and Cy3-conjugated anti-mouse (715-165-150, AB_2340813, 1 : 400) secondary antibodies raised in donkey were purchased from Jackson ImmunoResearch Lab (West Grove, PA). Coverslips were mounted with an antifading media with DAPI (Vectashield Hardset, Vector Laboratories).

For quantitative analysis of immunohistochemistry for IB4, CTB, CGRP, and synaptophysin or their overlaps, subsets of 50 sections of 500 serial cross sections (20 *μ*m thick) cut from 1 cm long spinal cord segments at T7 and T13 levels were subjected to confocal microscopy using a laser scanning microscope system (Olympus FV-300). Digital images (1024 × 1024 pixels) were taken with a 40x objective lens centered at superficial laminae I-II for IB4 and CGRP and laminae III-V for CTB in the lateral dorsal horn areas (317.44 × 317.44 *μ*m) that suffice to cover the entire projections of DCN IB4^+^ C fibers or CTB^+^ A fibers [20, 22]. In each section, a single confocal image was sampled at the mid *z*-plane at each laser wavelength channel. This single *z*-plane sampling has been consistently used for the quantification of immunohistochemical labeling in our previous studies [20, 22]. For a consistent quantification, all sets of sections were labeled with secondary antibodies from the same lots and images were obtained with the identical setting at the confocal microscope including pinhole size, laser intensity, digital gain, and exposure time.

### 2.8. Central Projection of DCN A and C Fibers

Immunoreactive areas of IB4^+^ C fibers and CTB^+^ A fibers were measured on confocal images of T7 and T 13 dorsal horns. Using ImageJ software (NIH, Bethesda, MD), images were converted to 8-bit black and white images and immunoreactive areas were measured as previously carried out [20, 22–25]. Threshold function in ImageJ creates red masks on pixels brighter than the choice of gray intensity (0-255) on the histogram. A threshold gray value was determined at the gray intensity in which red masks cover the entire immunoreactive areas. Pixels were selected at threshold gray values, and areas of selected pixels were measured as immunoreactive areas. All 50 serial sections were used to identify the dorsal root entry zone (DREZ) where the most labeled axon fibers were found and the rostral-caudal ends of each afferent projection where no immunoreactivity was appeared. As our previous data on sections at every 200 *μ*m in normal animals showed no significant fluctuation at the 1 mm distance [20], data were shown on sections with an interval of a millimeter from the DREZ in this report.

Selected pixels were saved as a separate image. Neighboring, selected pixels were outlined as particles for better visibility on spinal cord diagrams to analyze their spatial distribution in terms of laminar, medial/lateral, and dorsal/ventral locations. Reference lines of superficial laminae I-IV were identified based on the cytoarchitecture (DAPI nuclear counterstaining) and VGLUT1 distribution pattern in the dorsal horn [20, 26, 27] and copied to the same location on the particle outline images. Particles with laminar reference lines were then overlaid on representative dorsal horn diagrams aligning to edges of the lateral dorsal horn and the spinal cord tissue and to laminar reference lines. Consistent with our previous studies [20], a circle was drawn small as possible to encompass all particles and the circle area was measured for projection field areas. Density of DCN afferent projections was determined as immunoreactive axon areas (pixel areas) divided by projection field areas (circular areas).

### 2.9. Estimation of Putative Synaptic Terminal of DCN Afferents

Synaptic termination of DCN A and C fibers was estimated using a synaptic vesicle molecule, synaptophysin, that represents central terminations for both A and C fibers in the dorsal horn [22]. Other subsets of 50 serial sections from the same rats with axon tracer injections were subjected to double immunohistochemistry, i.e., IB4/synaptophysin or CTB/synaptophysin. Confocal images were taken at the same dorsal horn areas where immunoreactive areas were measured. The color threshold function in ImageJ was utilized to detect double labeling as established in our previous studies [20, 22]. Threshold values were determined as described above and applied for each labeling in separate color channels, for instance, CTB in green and synaptophysin in red. Both channels were combined in a stack to measure immunoreactive areas of color pixels that were positive for both labeling (yellow). Percent termination was calculated as percentage of double labeled areas in each axon tracer area on each section examined and averaged across locations at an interval of 1 mm.

### 2.10. CGRP Projections in the Dorsal Horn

CGRP was double labeled with IB4 on adjacent serial sections from the same animals in which the IB4^+^ C fiber projections were examined. As CGRP labeling was not DCN-specific, CGRP^+^ immunoreactive areas were selectively quantified in a randomly chosen mediolateral area where DCN IB4^+^ C fibers project [20]. A rectangular region of interest (ROI, 55,960 *μ*m^2^) was defined to include laminae I-II areas as wide as the maximal lateral distribution of DCN IB4^+^ C fibers determined in both T7 and T13 dorsal horns. An immunoreactive area for CGRP and percent colabeling between CGRP and IB4 were measured within the ROI based on the color thresholding as described above.

### 2.11. Statistical Analysis

Statistical analysis was performed in MATLAB or Prism software (v8, GraphPad Software, San Diego, CA). One sample *t*-test was used to select significant percent changes of each temporal (early or late) CTM reflex response in animals with SCI from normal animals. A two-tailed, unpaired *t*-test was used to compare mean values between two groups. Welch's correction was applied to the *t*-test when groups with unequal variances were compared. The significance of *t*-test was supplemented with, at least, a large (Cohen's *D* > 0.8) or a very large (*D* > 1.2) effect size [28]. The combined qualification of *t*-test and effect size was used to show effective changes made by the main effect of injury in independent physiological (early vs. late responses) and anatomical (A vs. C fibers) assessment groups and qualitatively compare those effective changes across different animal groups. For multiple pairwise comparisons between more than two variables (e.g., A/C fibers, spinal levels, sides, and injury) within each assessment group, the analysis of variance (ANOVA) test was used with Tukey's post hoc test. Correlation between two variables was determined by the Pearson correlation coefficient (Pearson's *r*) of a linear regression analysis. The threshold of significant probability was 95% (*p* < 0.05).

## 3. Results

### 3.1. Hemisection SCI Produces Cutaneous Nociceptive Hyperreflexia

**Figure 2:**
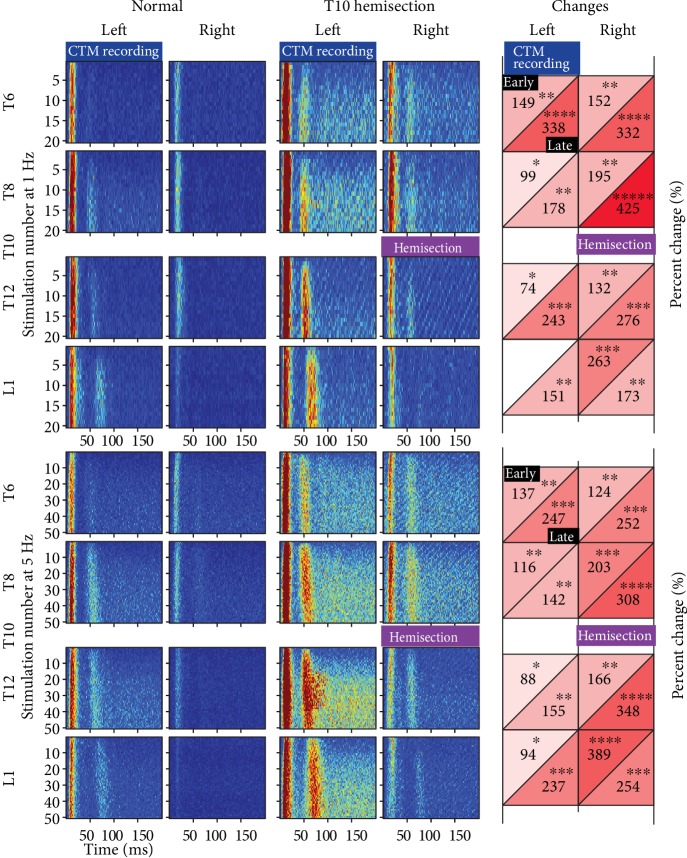
False color plots of CTM neurograms and percent changes 6 weeks after T10 hemisection spinal cord injury. CTM neurograms were recorded with 20 (1 Hz, top 4 plots) or 50 (5 Hz, bottom 4 plots) stimulations at a DCN level (either T6, T8, T12, or L1) at either ipsilateral (left) or contralateral (right) side to the side of CTM recoding (blue boxes). Each row (stimulation number) within each plot represents a color-scaled CTM response (see Figure 1) averaged in normal (*n* = 8) and injured (*n* = 7) animals for 200 milliseconds (ms) from each stimulation onset. The first stimulation in the train is the top row, and the last stimulation is the bottom row within each plot. Each box on the left diagrams (“changes”) displays only significant percent changes of early and late responses (see time windows in Figure 1) in animals with T10 hemisection injury (purple boxes) relative to normal controls (one sample *t*-test, *p* < 0.05 with large effect size, Cohen's *D* > 0.8) at each spinal level. Red color of boxes represents significant increase where darker red means greater extent of increases than lighter red, and the number of asterisks reflects the darkness of red color for black and white prints. There was no decreased response after SCI.

In normal uninjured rats, we have previously shown that CTM reflex responses varied by DCN stimulations with different stimulation frequencies at different spinal levels and sides (ipsi- vs. contralateral) [21]. Consistently, stimulations to DCNs ipsilateral to the CTM neurogram recording site, i.e., the left side of the rats, evoked both early and late reflex responses (Figure 1(b)). The contralateral DCN stimulations were able to evoke both early and late responses, but the size of the responses was noticeably smaller than those responses to stimulations on ipsilateral DCNs (Figure 2).

After T10 hemisection SCI, robust increases were found in the size of both early and late responses to stimulations at both ipsilateral and contralateral DCNs at both above and below the levels of injury as shown in the pseudocolor plots (Figure 2). Early and late responses in animals with SCI were normalized to those responses in uninjured normal control animals. In Figure 2, one sample *t*-test qualified almost all changes of each response after SCI (*p* < 0.05) across different spinal levels and different stimulation frequencies (1 and 5 Hz), and these selected changes also demonstrated large effect size (Cohen's *D* > 0.8). ANOVA showed significant differences between changes of temporal (early vs. late) responses evoked at different (ipsi- vs. contralateral) sides (*F* = 5.31, *p* = 0.01 at 1 Hz and *F* = 4.66, *p* = 0.02 at 5 Hz) suggesting the effect of stimulation sides on changes of each CTM reflex response after injury. However, multiple pairwise comparisons demonstrated no significant differences between matching temporal responses from each side (e.g., right early/late vs. left early/late) across spinal levels at both 1 and 5 Hz (Tukey's post hoc test, *p* > 0.16). Collectively, T10 hemisection SCI produced cutaneous nociceptive hyperreflexia measured with significantly increased size of both early and late responses of the evoked CTM reflex at both sides of injury both above and below the injury site at T6, T8, T12, and L1.

### 3.2. DCN Afferents Sprout Following Hemisection SCI

**Figure 3:**
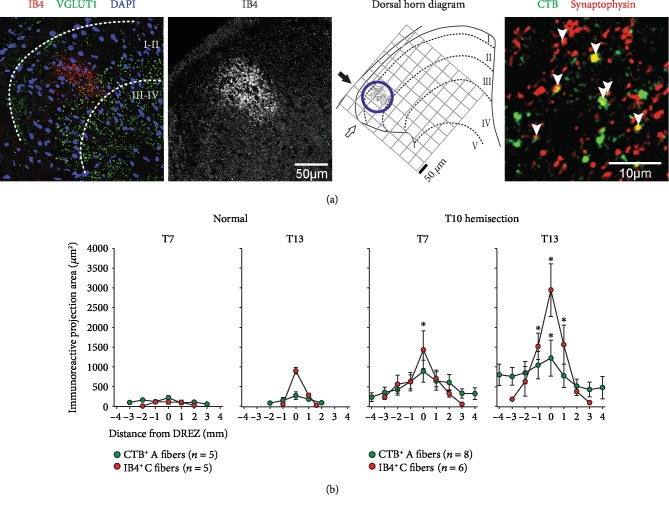
Central projection analysis of DCN afferents labeled with peripheral CTB and IB4 tracer injections. (a) A representative confocal image shows IB4^+^ C fiber projection in T13 dorsal horn. The IB4^+^ immunoreactive area was measured on a gray scale image and outlined as particles. Images with outlined particles were aligned to the edges of dorsal horn (white arrow) and spinal cord (black arrow) on a diagram to reconstruct spatial distribution as shown in Figure 4. A circle was drawn to encompass all particles and measured as a projection field area (blue circle). Synaptophysin was colabeled with either CTB or IB4 to estimate number of putative synaptic terminals of DCN A and C fibers. (b) Immunoreactive areas of CTB^+^ A fibers and IB4^+^ C fibers were measured on serial sections at every 1 mm both rostral (-) and caudal (+) to the dorsal root entry zone (DREZ) at T7 and T13 spinal cord segments (mean value ± standard error of the mean). Both CTB^+^ A fibers and IB4^+^ C fibers showed no significant differences between ipsi- and contralateral sides in subsets of injured rats, and data from both sides were combined for each afferent at each segment. Asterisks indicate significant differences from normal controls at given location (ANOVA with Tukey's multiple comparisons, *p* < 0.05). Images in (a) and immunoreactive area data for normal animals in (b) were adopted and modified from our previous study [20].

**Figure 4:**
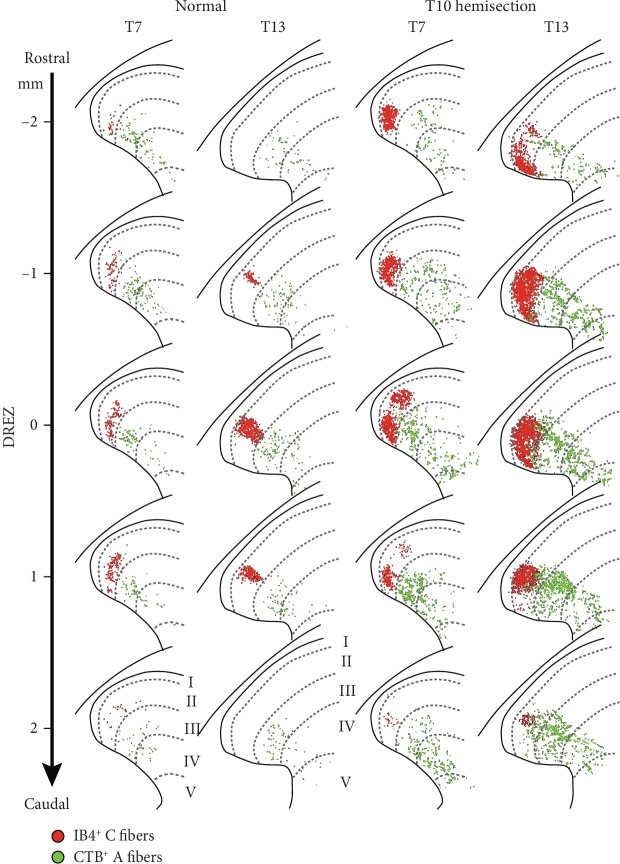
Spatial distribution of A and C fiber central projections from DCNs in the spinal cord of an uninjured normal control and an injured animal 6 weeks after T10 hemisection. CTB^+^ A fibers (green dots) and IB4^+^ C fibers (red dots) from T7 and T13 DCNs were identified as immunoreactive particles and overlaid on dorsal horn diagrams to show distribution profiles in terms of laminar, medial/lateral, and dorsal/ventral locations (see also Figure 3(a)). Representative projections were displayed on rostral (-) and caudal (+) cross sections to the dorsal root entry zone (DREZ) of the 4 mm spinal cord tissue block analyzed from a normal (left two columns) and an injured (right two columns) animal. Images for the normal control animal were adopted and modified from the previous publication [20].

Central projections of DCN A and C fibers have been previously characterized in T7 and T13 spinal cords in normal uninjured rats [20]. To investigate changes in nociceptive afferent projections after T10 hemisection SCI, T7 and T13 DCNs were injected with CTB and IB4 to label A and C fibers, respectively. Immunoreactive areas of CTB^+^ A and IB4^+^ C fibers were quantified on serial spinal cord sections. The section with the most labeled axons was defined as the dorsal horn entry zone (DREZ) at each T7 and T13 level (Figures 3(b) and 4). An axon tracer (CTB or IB4) was injected into DCNs ipsilateral to hemisection and the other tracer into contralateral DCNs in a subset of animals, while the other subset of animals received those injections at the opposite side, to examine differences of each fiber projection between each side of hemisection SCI. An ANOVA test rejected the effect of different sides (*F* = 0.10, *p* = 0.10 for A fibers and *F* = 0.26, *p* = 0.95 for C fibers at T7; *F* = 0.67, *p* = 0.71 for A fibers and *F* = 0.67, *p* = 0.67 for C fibers at T13) and showed no significant differences between means from each side at each distance from DREZ at each spinal level (Tukey's post hoc test, *p* > 0.15), so quantitative data for each axon label from both sides were combined at each T7 and T13 level (Figure 3(b)).

**Figure 5:**
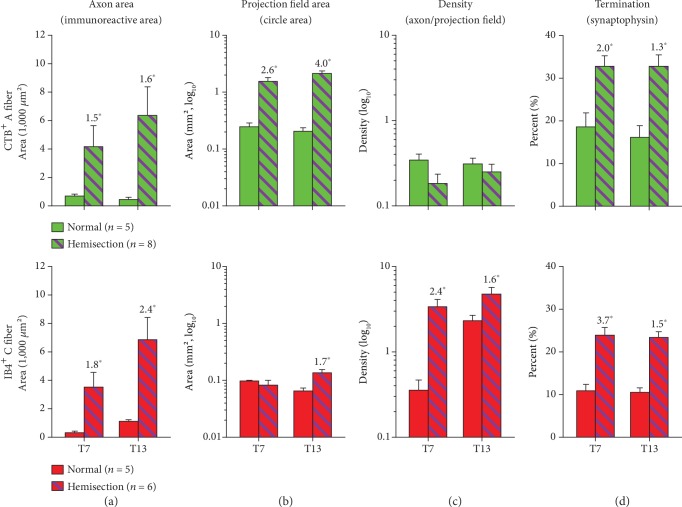
Changes in central projection of DCN afferents 6 weeks after hemisection SCI. (a–c) Projections of IB4^+^ C and CTB^+^ A fibers were analyzed in T7 and T13 dorsal horn in terms of the axon area (immunoreactive axon area, Figure 3(b)), projection field area (circle area, Figure 3(a)), and density (axon/projection field). (d) Synaptic termination of DCN afferents was assessed as percent colabeling of each axon label with synaptophysin. Shown are mean values with standard error of the mean. Numbers above error bars indicate Cohen's *D* effect size only for significant changes after hemisection SCI (normal vs. hemisection, ∗) at each level (*t*-test, *p* < 0.05). There was no segmental difference in each (normal or hemisection) group in (a)–(d) (ANOVA with Tukey's multiple comparison).

At 6 weeks post SCI, immunoreactive areas of both CTB^+^ A and IB4^+^ C fibers were drastically increased at rostral-caudal locations centered at DREZs in both T7 and T13 spinal levels (Figures 3(b) and 4). ANOVA confirmed the significant effect of SCI on the overall increases of both CTB^+^ A fibers (*F* = 42.23, *p* < 0.0001) and IB4^+^ C fibers (*F* = 7.76, *p* = 0.04) across T7 and T13 locations compared to normal controls. The most noticeable change was the appearance of peaks at DREZs for both CTB^+^ A and IB4^+^ C fibers even at a T7 spinal level where no clear peaks were found in normal animals (Figure 3(b)). Tukey's multiple comparison test showed significant increases of CTB^+^ A fibers at T13 (*p* = 0.04) and IB4^+^ C fibers at T7 (*p* < 0.0001) and T13 (*p* = 0.0002) at DREZs after SCI. The rostral-caudal distributions of both CTB^+^ A and IB4^+^ C fibers were extended at least 1 mm to each direction from the DREZ. Projections of A fibers extended from ±3 mm to ±4 mm in T7 and from ±2 mm to ±4 mm in T13 while C fiber projections extended from ±2 mm or less to ±3 mm in both T7 and T13 at 6 weeks after SCI (Figure 3(b)). To summarize central projections of CTB^+^ A and IB4^+^ C fibers, immunoreactive areas on dorsal horn sections were summed across all rostral-caudal locations (Figure 5(a)). Total summed areas of both CTB^+^ A and IB4^+^ C fibers were significantly increased (two-tailed, unpaired *t*-test, *p* < 0.05) showing very large effect sizes (Cohen's *D* > 1.2 [28]) at both T7 and T13. These data demonstrated the central sprouting of both DCN A and C fibers both above and below the T10 hemisection SCI that also produced the nociceptive hyperreflexia evidenced by significant increases in both early and late responses of the CTM neurograms on both sides of animals both above and below that injury.

### 3.3. Hemisection SCI Changes Projection Patterns of DCN Afferents

Changes in central projection patterns of DCN A and C fibers were investigated in T7 and T13 dorsal horns in rats with T10 hemisection SCI. The increases in immunoreactive areas after SCI (Figure 5(a)) were related to the expansion of dorsal horn areas in which DCN afferents projected (Figure 4). To analyze projection field areas, the size of circles that encompassed CTB^+^ A or IB4^+^ C fibers on the dorsal horn was measured (Figure 5(b)). DCN A fibers significantly expanded their projection field at both T7 and T13 following hemisection, whereas C fiber projection field enlarged only at T13 (two-tailed, unpaired *t*-test, *p* < 0.05, Cohen's *D* > 1.2).

To understand the relationship between the axon sprouting and their spatial distribution in the dorsal horn, axon density in projection field was analyzed (Figure 5(c)). The increased projections of DCN A fibers dispersed in the significantly expanded projection fields resulting in no changes of axon density after SCI. In contrast, DCN C fibers sprouted in relatively comparable projection field areas giving rise to significantly increased density at both T7 and T13 (two-tailed, unpaired *t*-test, *p* < 0.05, Cohen's *D* > 1.2). These data demonstrated differential sprouting patterns of nociceptive afferent types following T10 hemisection SCI such that C fibers arborized within their projection field, but A fibers sprouted into dorsal horn areas where DCN A fibers do not innervate in normal animals.

### 3.4. Synaptic Terminations of Sprouting Cutaneous Afferents

To determine whether the sprouting DCN afferents increased synaptic inputs of the CTM reflex after SCI, putative synaptic terminals of CTB^+^ A and IB4^+^ C fibers were estimated using a general presynaptic marker, synaptophysin, in T7 and T13 dorsal horns [22]. Areas that were double labeled with an axon tracer (CTB or IB4) and synaptophysin were measured on serial sections at every millimeter from DREZ as in Figure 3(b). Double labeled areas were normalized to axon areas of each axon tracer and averaged across all locations (Figure 5(d)). The percent colabeling significantly increased in both CTB^+^ A and IB4^+^ C fibers at both T7 and T13 spinal levels after SCI (two-tailed, unpaired *t*-test, *p* < 0.05, Cohen's *D* > 1.2). As the percent colabeling indicates numbers of putative synaptic terminals per axon, these increased colabeling ratios demonstrated that the sprouting DCN axons after SCI not only increased their central projections but also formed synaptic terminations to the greater extent to which those afferents did in normal uninjured conditions. The results are consistent with the hypothesis that cutaneous afferents enhance their synaptic inputs to the nociceptive spinal signal processing contributing to the hyperreflexia seen in the CTM reflex after T10 hemisection SCI.

### 3.5. CGRP Immunoreactive Areas Do Not Increase in the Dorsal Horn

**Figure 6:**
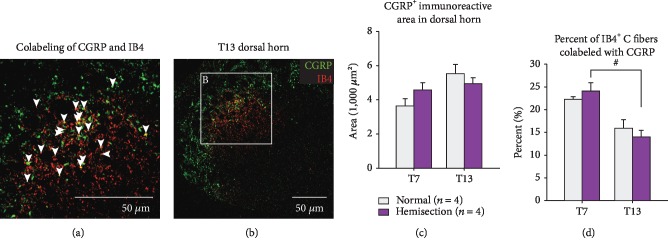
CGRP expressing C fibers in T7 and T13 spinal dorsal horns and overlapping of CGRP expression in IB4^+^ DCN C fibers after T10 hemisection. (a) A representative confocal image shows immunohistochemistry for IB4 injected into DCN C fibers and for CGRP at a T13 dorsal horn. (b) High power view of the representative image demonstrates CGRP expression in IB4^+^ C fibers at the superficial dorsal horn (arrows). (c, d) Immunoreactive areas of CGRP^+^ peptidergic C fibers (c) and percent colabelings of CGRP in IB4^+^ C fibers (d) were measured in T7 and T13 dorsal horns in normal and hemisection SCI animals. Bar graphs show mean values with standard errors of the mean. Crosshatch displays significant segmental differences between T7 and T13 (ANOVA with Tukey's multiple comparison, *p* < 0.05).

Due to the lack of a transganglionic tracer for peptidergic C fibers, immunohistochemistry for CGRP on dorsal horn sections was used to estimate the central projection of DCN peptidergic C fibers as previously done [20]. Regions of interest were defined in lamina I-II corresponding to the medial/lateral locations where DCN-specific IB4^+^ C fibers projected (Figures 6(a) and 6(b)). CGRP labeling was found in lamina I and in the outer layer of lamina II. There were no noticeable changes of laminar distributions of CGRP labeling after SCI. Immunoreactive areas of CGRP in the examined dorsal horn regions were not changed both at T7 and T13 following T10 hemisection SCI when compared to uninjured normal animals (Figure 6(c), ANOVA, *F* = 3.249, *p* = 0.06). Tukey's multiple comparisons showed no segmental difference in both normal and hemisection groups (*p* > 0.47).

As we have previously shown that approximately 10% of cell bodies of IB4^+^ C fibers also expressed CGRP in the DRGs [20], changes in overlap between IB4 and CGRP after SCI may affect the CGRP^+^ dorsal horn areas. Areas of double labeling with CGRP and IB4 (Figure 6(b)) were measured, and percent colabeling to DCN IB4^+^ C fibers was averaged across rostral-caudal locations in T7 and T13 spinal cords (Figure 6(d)). ANOVA (*F* = 9.803, *p* = 0.002) with Tukey's multiple comparisons showed no significant difference of percent colabeling in comparison between normal and injured animals (*p* > 0.81) but between T7 and T13 only after SCI (*p* < 0.05).

### 3.6. Injury Severity Correlates to the Extent of Afferent Sprouting

**Figure 7:**
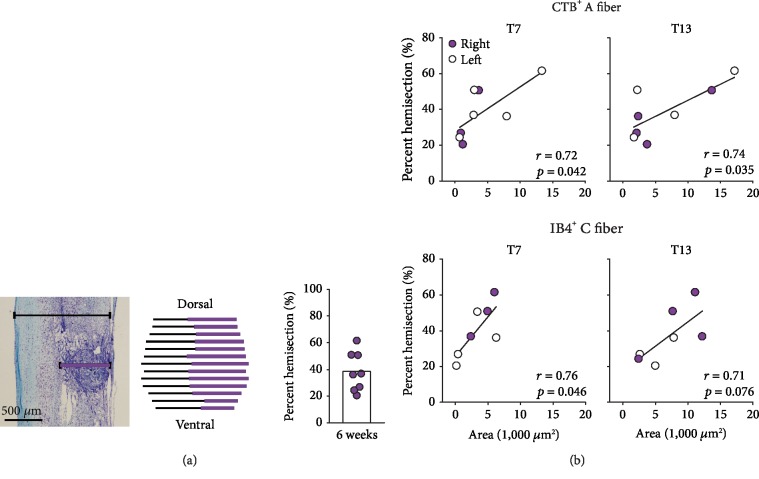
Percent hemisection injury and correlation between injury severity and DCN afferent sprouting. (a) Size of damaged tissue was measured on longitudinal sections stained with Luxol fast blue and Cresyl violet 6 weeks after T10 hemisection SCI. Percent hemisection injury was calculated as the maximal, lateral width of the damaged tissue (the length of the purple line) divided by the lateral width of the entire spinal cord tissue (the length of the black line) times 100. This was averaged across serial longitudinal sections at every 100 *μ*m in individual animals as shown in a dorsal-ventral reconstruction of a representative animal. (b) Summed immunoreactive axon areas for CTB^+^ A fibers and IB4^+^ C fibers (Figure 5) labeled on right (injured, purple circle) or left (noninjured, white circle) side of animals were plotted against the measured percent injury in each animal at T7 and T13 levels. Linear regression was analyzed between the percent injuries and the summed axon areas from both sides shown as Pearson's *r* and *p* values.

Chronically injured spinal cord tissues typically form the prominent fibroglial scar that consists of fibroblasts, dense extracellular matrix (ECM), and astroglial limitans. To evaluate injury severity, the size of injury (i.e., the size of scar tissue) was measured on serial longitudinal sections at T10 spinal cords in individual animals with SCI. At 6 weeks after injury, Cresyl violet staining showed a dense fibrotic core tissue surrounded by injury-specific, astroglial cytoarchitecture (Figure 7(a)). There was an approximate average of 40% hemisection injury across 8 rats. Linear regression analysis indicated a positive correlation between percent hemisection injury and immunoreactive areas for both CTB^+^ A and IB4^+^ C fibers at both T7 and T13 (Figure 7(b)). This demonstrated that the more severe SCI induced the greater intraspinal sprouting of cutaneous specific afferents above and below the level of injury (Figure 7) which may contribute to nociceptive hyperreflexia comprehensively evoked by stimulations throughout lumbar thoracic DCNs (Figure 2).

## 4. Discussion

**Figure 8:**
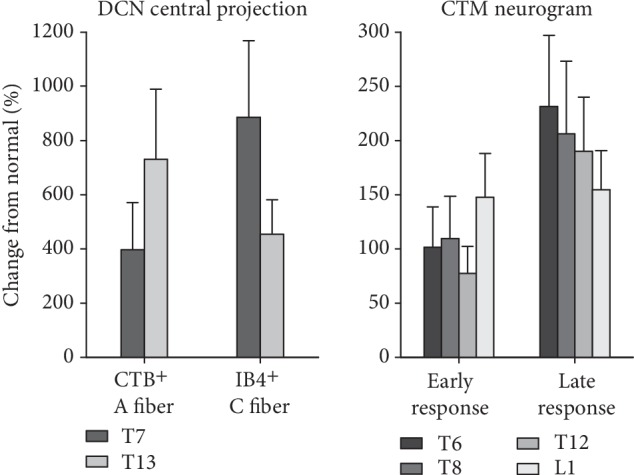
Comparison between DCN afferent sprouting and CTM neurogram responses at different spinal segmental levels after 6 weeks following T10 hemisection SCI. Changes in CTB^+^ A fiber and IB4^+^ C fiber projections from T7 and T13 DCNs were calculated relative to uninjured normal animals (axon areas in Figure 5). Changes from normal animals in early and late responses of CTM neurograms evoked by stimulations at different DCN levels (T6, T8, T12, and L1; left column in Figure 2) were averaged across both sides (left and right) and both stimulation frequencies (1 and 5 Hz).

This report demonstrated that T10 hemisection SCI produced cutaneously evoked nociceptive hyperreflexia at 6 weeks both above (T6 and T8) and below (T12 and L1) the injury. We have found that central projections of cutaneous afferents, i.e., CTB^+^ A and IB4^+^ C fibers significantly increased both above (T7) and below (T13) the injury at T10. In addition, these sprouting DCN afferents expanded their projection territory and increased their synaptic termination in the dorsal horn. These data support the hypothesis that SCI induces sprouting of DCN afferents away from the injury and increased the size of evoked CTM reflex responses resulting in nociceptive hyperreflexia. Due to potential damage of DCN afferents by axon tracer injections, we used different groups of animals for anatomical or electrophysiological experiments. Therefore, we could not directly correlate the extent of sprouting for different DCN afferent subtypes, A vs. C fibers, with changes of different temporal parts of CTM reflex responses, early vs. late responses, respectively, in each individual animal. Averaged changes of afferent sprouting were compared to summarize changes of CTM neurogram responses at corresponding spinal levels (Figure 8). DCN A fiber sprouting tended to be greater caudally at T13 than those changes at T7. Despite the lack of clear rostral-caudal tendency, changes of CTM early responses to a caudal DCN stimulations at L1 are greater than those changes to rostral DCN stimulations at T6. Interestingly, DCN C fibers and CTM late responses showed an opposite tendency being progressively decreasing toward caudal DCNs. These rostral-caudal patterns of changes after SCI support the association of independently observed significant changes in DCN afferent projections with evoked nociceptive hyperreflexia in, at least, late component of the CTM reflex above and below the level of the injury.

Central changes of primary nociceptive neurons following SCI, mostly reported as sprouting new branches in the dorsal horn, are considered a chronic driver of nociceptive hyperreflexia by potentially producing excessive nociceptive input to the spinal pain signal processing, so-called central sensitization [29]. The mechanism that activates intrinsic growth capability of nociceptive afferents after SCI has been sought in an altered neurotrophic environment. The blockade of nerve growth factor (NGF) which is known to promote growth of trk-A bearing small diameter nociceptive afferents, A*δ* and peptidergic C fibers, significantly reduced central sprouting of CGRP expressing C fibers following SCI [7]. A recent study has shown that early forced exercise in rats with cervical SCI neutralized decrease of glial cell line-derived neurotrophic factor (GDNF), reduced nonpeptidergic C fiber sprouting, and prevented the development of allodynia in below the level of injury [11]. The altered neurotrophic environment induces expression of growth associated molecules such as growth associated protein 43 (GAP43) that have been shown to colocalize with sprouting CGRP projections in the dorsal horn [30, 31].

Inflammatory responses have been known to be central to the neurotrophic supplements that trigger the intrinsic growth of pain afferents following SCI. Moreover, widespread, long-lasting inflammation across the injured spinal cord is responsible for sprouting of nociceptive afferents away from the injury, which may develop pain syndromes at unaffected body parts in SCI patients. The first reported sprouting of nociceptive C fibers has been observed in lower lumbar segments in association with compensatory increase of muscle afferent inputs to recovered postural reflexes after thoracic hemisection in cats [31]. Indeed, activation of microglia and astrocytes has been reported at cervical (C6/7) and lumbar (L4/5) segments after T10 contusion SCI in rats and the microglial activation lasted at least 6 months after that injury [32]. The SCI-induced inflammatory responses are systemic and affect the peripheral nervous system as well. Microglial and astrocytic activation after T10 contusion SCI in rats resulted in neuron damages in the cervical DRGs, induced central sensitization of forelimb nociceptive afferents, i.e., enhanced spontaneous activity contributing to mechanical allodynia and thermal hyperalgesia [33]. These data suggest that persistent inflammatory activity for 6 weeks after T10 hemisection SCI induces sprouting of DCN A and C fibers at T7 and T13 developing nociceptive hyperreflexia as seen as increased CTM reflex responses at those spinal levels.

The intraspinal sprouting of DCN afferents appears to be independent from the loss of intersegmental connection of the CTM reflex pathway. As the CTM reflex could be evoked by stimulating one side of bilateral DCNs on both sides at each spinal level (Figures 1 and 2), we made hemisection SCI to disconnect ascending propriospinal interneurons only at the side of injury below the level of injury (see Figure 1(a)), which may create differences between both sides. However, DCN A and C fibers sprouted (Figures 3(b) and 5(a)) and increased their synaptic terminations (Figure 5(d)) at both sides contributing to significant increases of CTM reflex responses evoked at both ipsilateral and contralateral DCN stimulations below the injury (Figure 2). This suggests that central sprouting of nociceptive afferents away from SCI may have resulted from widespread inflammation rather than the loss of intersegmental interneurons. In addition, it is interesting that the loss of supraspinal inhibition below the injury at the hemisection side demonstrated no noticeable differences in changes of CTM reflex responses when compared to those changes above the level of injury. This may be due to the fact that the supraspinal interneurons do not play a role on the evoked CTM reflex responses or that the damaged interneurons after hemisection die back and removed their inhibition to rostral segments above the injury as well. A novel finding in the current studies with hemisection SCI was that CTM neurogram responses to contralateral DCN stimulations at the side of injury were increased both above and below the level of injury. This provided a critical clue for understanding the interneuronal organization of the CTM reflex. As there was no projection of CTB^+^ A fibers or IB4^+^ C fibers to the contralateral dorsal horn in normal [20] and hemisection rats in the current studies, the contralateral CTM response requires a decussation of spinal interneurons either at the same segmental level or at cervicothoracic junction where the CTM motor nucleus locates (Figure 1(a)). The contralateral CTM responses below the level of injury clearly demonstrated the existence of at-level decussation. The increased contralateral responses above and below the injury suggests that the cervicothoracic decussation plays no or very limited roles on the contralateral CTM responses. Due to the relatively short latency of contralateral responses compared to ipsilateral responses (Figure 2), there are likely a single interneuronal connection or a few synaptic relays in the at-level decussation. Collectively, the current knowledge can be summarized such that interneurons of the CTM reflex form a small number of synaptic relays, decussate at the level of DCN projections, ascend along the ventral half of the lateral funiculus [15], and terminate onto the motoneurons in the cervicothoracic nucleus [18].

One of the possible mechanisms for the increased CTM reflex responses would be the loss of supraspinal inhibition following SCI. Although not much is known yet, there may be three supraspinal inhibitory connections in the CTM reflex pathway: one on the cervicothoracic motoneurons, another on ascending propriospinal interneurons at each DCN level, and the last on the interneurons that decussate from the contralateral side at each level (Figure 1(a)). The loss of inhibition on the CTM motoneurons and ipsilateral interneurons at T7 and T13 would not be relevant to contralateral hemisection SCI at T10. Hemisection contralateral to the recording site in the current studies removed the potential supraspinal inhibitory connection to the decussating contralateral interneurons below the level of injury. The loss of contralateral inhibition seems to generate no significant differences as CTM responses to contralateral DCN stimulations were increased both above and below the level of injury. Therefore, the nociceptive hyperreflexia of the CTM reflex may not be a result of loss of inhibition.

A remarkable alteration in the pseudocolor plots of the CTM neurograms after SCI was a prolonged elevation of background activities over the phase of recovery to the baseline (Figure 2). Increased background activity was seen with repeated stimulation even in uninjured normal animals, for instance, at T8 and T12 DCN stimulation on the left side. This phenomenon was profound with almost all DCN stimulations in animals with SCI but less noticeable on the contralateral (hemisection) side below the level of injury. The facilitation was found over the course of stimulation trains and with increasing stimulation frequency, indicating the “windup” phenomenon [34]. This windup effect of the CTM reflex supports the idea of the central sensitization of nociceptive afferent inputs in the CTM reflex after SCI.

The topographical representation of different primary afferents has been extensively demonstrated in the dorsal horn using intracellular injections of labels to electrophysiologically identified, individual DRG neurons [35, 36]. These studies commonly demonstrated that projecting primary afferents enter the spinal cord through the DREZ, branch axons in the rostral and caudal axis, and arborize in designated areas in the dorsal horn depending on afferent types. We have previously found that IB4^+^ DCN C fibers exclusively projected in lamina II and CTB^+^ DCN A fibers dispersed in laminae III-V at the lateral dorsal horn [20]. After hemisection SCI, both DCN A and C fibers elongated their rostral-caudal distribution (Figure 3(b)). In terms of laminar distribution, IB4^+^ C fibers increased the density within their projection field whereas CTB^+^ A fibers expanded their projection field. These propose a hypothesis that cutaneous A and C fibers have differential contributions to nociceptive hyperreflexia after SCI. The rostral-caudal expansion of both A and C fibers may result in the activation of additional CTM interneurons at neighboring spinal segments. Increased projection field areas of DCN A fibers indicate a potential recruitment of other interneurons (1) that form polysynaptic relay connections to the CTM interneurons, (2) that modulate inhibitory mechanisms of nociceptive signal processing (e.g., gate control theory) [37, 38], and (3) that are not involved in the CTM reflex creating new functions (e.g., autonomic responses).

Numerous studies have reported sprouting of CGRP expressing afferents into inappropriate laminae in association with altered somatosensory function after SCI [7, 8, 10, 12, 30]. For instance, CGRP sprouting in laminae III-IV that was related with touch, pressure, and kinesthesia was associated with the development of chronic pain syndromes in cervical and lumbar segments after thoracic hemisection SCI [30]. CGRP^+^ afferents that sprout deeper like in lamina V-VII appear to recruit preganglionic sympathetic neurons in the intermediolateral nucleus in animals with autonomic dysreflexia following SCI [8, 10, 12]. However, increased immunoreactivity for CGRP in the dorsal horn seems not to represent nociceptive hyperreflexia after SCI. Compression SCI models that developed chronic or neuropathic pain reported no CGRP sprouting but decreased immunoreactivity in both superficial laminae (I-II) and deeper laminae (III-V) rostrally at T10-T12 after T13 injury [39, 40]. We also found no changes of CGRP immunoreactive areas in the superficial dorsal horn at T7 and T13 at 6 weeks after T10 hemisection. This might be simply due to different injury types (hemisection vs. compression injury) and different spinal levels (thoracic vs. cervical/lumbar segment). Another possible explanation is that CGRP immunoactivity in the dorsal horn is not specific for nociceptive, peptidergic C fibers but includes afferent subpopulations projecting from nonnociceptive peripheral nerves. Indeed, CGRP immunoreactivity in the dorsal horn has been found in A*β*, A*δ*, and C fiber afferents that were electrophysiologically identified and labeled in the DRG [41]. These suggest that CGRP sprouting in superficial laminae may not be necessary to create, at least, cutaneously evoked nociceptive hyperreflexia after hemisection SCI.

Interestingly, the overlap of IB4 labeling with CGRP immunohistochemistry in the dorsal horn became significantly different between T7 and T13 after SCI. However, none of the changes in either IB4-labeled or CGRP immunoreactive area alone support the segmental difference of overlap. The only possible explanation, based on our current knowledge of significantly increased IB4^+^ projections but no changes of CGRP^+^ immunoreactive areas at both T7 and T13, would be that more CGRP expressing cells that were not positive for IB4 in normal condition bound IB4 after SCI at T7 than those cells at T13. Along with no change of the projection field area, i.e., remarkably increased projection density of C fibers only at T7, it would require further investigation to analyze detailed cellular profile changes of C fiber subpopulations in different levels of SCI.

## 5. Conclusions

Despite ongoing incomplete understanding of the relationship between anatomy and physiology, our data collectively demonstrated that SCI induced sprouting of DCN afferent subtypes both above and below the level of SCI depending on the injury severity, increased their synaptic terminations, and contributed to evoked nociceptive hyperreflexia of the CTM reflex. This report also demonstrated that the CTM reflex provides a useful SCI model in which anatomical and/or physiological plasticity can be measured to compare pathological changes to uninjured normal condition. Our interest extends to testing anti-inflammatory medications which may block sprouting of DCN afferents and neutralize nociceptive hyperreflexia following SCI.

## Data Availability

The data used to support the findings of this study are included within the article.
